# Crystallization of D-A Conjugated Polymers: A Review of Recent Research

**DOI:** 10.3390/polym14214612

**Published:** 2022-10-30

**Authors:** Yibo Hu, Xinxiu Cao, Hui Fan

**Affiliations:** 1 School of Materials Science and Engineering, Hunan University of Science and Technology, 2 Taoyuan Street, Xiangtan 411201, China; 2 Hunan Provincial Key Laboratory of Advanced Materials for New Energy Storage and Conversion, Hunan University of Science and Technology, 2 Taoyuan Street, Xiangtan 411201, China

**Keywords:** D-A conjugated polymers, polymer features, crystallization process, crystal structure, crystal morphology

## Abstract

D-A conjugated polymers are key materials for organic solar cells and organic thin-film transistors, and their film structure is one of the most important factors in determining device performance. The formation of film structure largely depends on the crystallization process, but the crystallization of D-A conjugated polymers is not well understood. In this review, we attempted to achieve a clearer understanding of the crystallization of D-A conjugated polymers. We first summarized the features of D-A conjugated polymers, which can affect their crystallization process. Then, the crystallization process of D-A conjugated polymers was discussed, including the possible chain conformations in the solution as well as the nucleation and growth processes. After that, the crystal structure of D-A conjugated polymers, including the molecular orientation and polymorphism, was reviewed. We proposed that the nucleation process and the orientation of the nuclei on the substrate are critical for the crystal structure. Finally, we summarized the possible crystal morphologies of D-A conjugated polymers and explained their formation process in terms of nucleation and growth processes. This review provides fundamental knowledge on how to manipulate the crystallization process of D-A conjugated polymers to regulate their film structure.

## 1. Introduction

Conjugated polymers are promising semiconductor materials for organic solar cells (OSCs) and organic thin-film transistors (OTFTs) due to their intrinsic advantages, including flexibility, light weight, solution processability and so on [1,2,3,4,5,6]. Over the past few decades, the development of conjugated polymers has undergone first generation materials such as polyacetylene, second generation materials such as poly(3-alkylthiophene)s (P3ATs) and now third generation materials such as donor–acceptor (D-A) conjugated polymers [7]. As the key layer of these devices, the structure of D-A conjugated polymer films, or blend films, is a critical factor for determining device performance [8,9,10,11,12,13,14,15,16,17]. However, the structure of D-A conjugated polymer films, or blend films, is very complex due to the multilevel structure in polymer materials.

**Figure 1:**
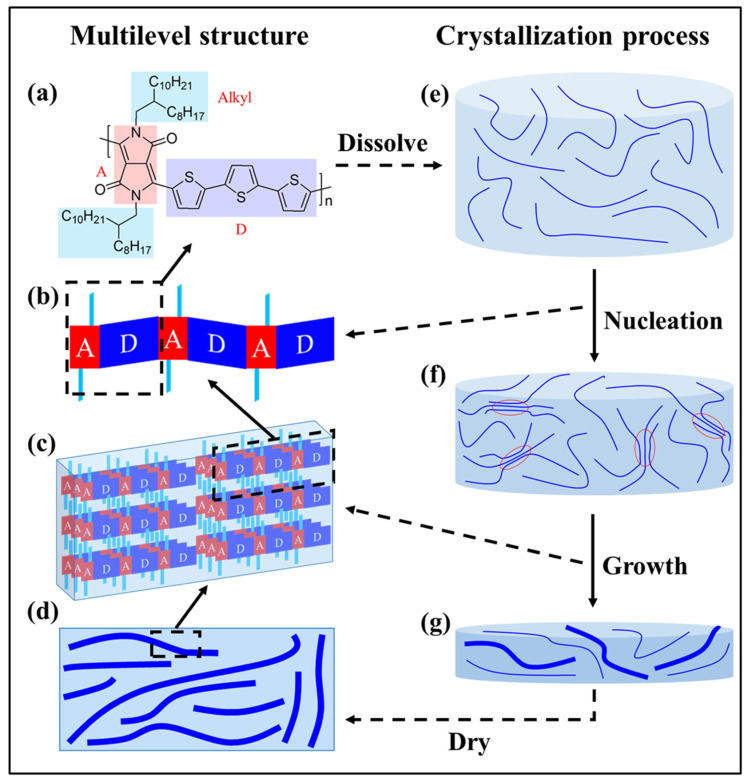
Schematic diagrams of the multilevel structure in D-A conjugated polymer films (**left**) and the typical crystallization process of D-A conjugated polymers (**right**). (**a**) represents the chemical structure of D-A conjugated polymers; (**b**) represents the chain conformation of D-A conjugated polymers; (**c**) represents the crystal structure of D-A conjugated polymers; (**d**) represents the film structure of D-A conjugated polymers; (**e**) represents D-A conjugated polymer solution; (**f**) represents the nucleation of D-A conjugated polymer crystals; and (**g**) represents the growth of D-A conjugated polymer crystals.

As shown in Figure 1, the structure of D-A conjugated polymer films, or blend films, can be divided into four levels [16,18]: (1) the short-range structure, including the chemical structure of the acceptor unit, the donor unit and the alkyl side chain; (2) the long-range structure, including the molecular weight, the molecular weight distribution and the conformation of the backbone and the side chain; (3) the crystal structure; and (4) the film structure. For OTFTs, D-A conjugated polymer films consist of crystalline domains and amorphous domains. Thus, the crystallization process of D-A conjugated polymers determines the resulting film structure. In the case of OSCs, blend films are composed of a polymer donor and a polymer or small molecular acceptor. In general, a three-phase model, consisting of a donor crystalline phase, an acceptor crystalline phase, and a mixed phase, is used to describe the structure of the blend films [19,20,21,22]. The formation of blend films undergoes a competition between the crystallization of the conjugated polymers and the phase separation of the donor and acceptor components [23,24,25]. No matter when the crystallization process occurs, it plays an important role in determining the structure of donor crystallites and acceptor crystallites. Therefore, understanding the crystallization process of D-A conjugated polymers is very important in manipulating the structure of D-A conjugated polymer films, or blend films.

The crystallization of D-A conjugated polymers generally occurs in a supersaturated solution driven by a decrease in the temperature or the evaporation of the solvent. As shown in Figure 1, conjugated polymers are first dissolved in the solvent to form a solution. Then, crystals emerge through the nucleation and growth processes. Finally, the structure of D-A conjugated polymer films, or blend films, is frozen when all the solvent has evaporated. The crystallization process could be regarded as a bridge linking the D-A conjugated polymers and their structure in the films. In this review, we attempted to provide a clearer understanding of the crystallization of D-A conjugated polymers based on the progress of recent research. We first discussed the fundamental features of D-A conjugated polymers from the perspective of crystallization. Then, we presented the current understanding of the crystallization process of D-A conjugated polymers, including the chain conformation in solutions as well as the nucleation and growth processes. We tried to associate the crystallization process of D-A conjugated polymers with the fundamental features of these polymers. Finally, we summarized the crystal structure and crystal morphology of D-A conjugated polymers. We also tried to associate them with the crystallization process and the fundamental features of D-A conjugated polymers. In addition, the synthesis of D-A conjugated polymers, the control of film morphology, and the relationship between film structure and device performance are also very important for the application of D-A conjugated polymers. The above topics were reviewed by other groups, and readers can refer to them [13,14,15,16,17,18,26,27,28,29,30,31,32,33,34].

## 2. The Features of D-A Conjugated Polymers

The chemical structure of D-A conjugated polymers consists of an alkyl side chain and a conjugated backbone formed by an alternating array of donor and acceptor units [35] as shown in Figure 1a. The backbone structure of D-A conjugated polymers is critical for their applications. The alternating array of donor and acceptor units results in a strong intermolecular charge-transfer interaction, making the energy levels of D-A conjugated polymers reasonably tuned [35,36]. The highly planar backbone and the strong π-π interaction between adjacent D-A subunits are beneficial to the intrachain and interchain charge transport, respectively [37,38,39]. The original idea of introducing the alkyl side chain was made to increase the solubility of conjugated polymers by increasing the interactions between the side chain and solvent or by decreasing the interactions between π-conjugated systems [40]. The forms of the alkyl side chain could be linear or branched [41]. Later, functional side chains for tuning the absorption, enhancing the polarity, promoting the ion conductivity and so on were developed [42,43,44,45]. Overall, the structure of D-A conjugated polymer chains is complex and distinctive, which gives them unique crystallization characteristics. The features of D-A conjugated polymers that will affect their crystallization process were discussed as follows.

### 2.1. The Regioregularity (RR) of D-A Conjugated Polymers 

**Figure 2:**
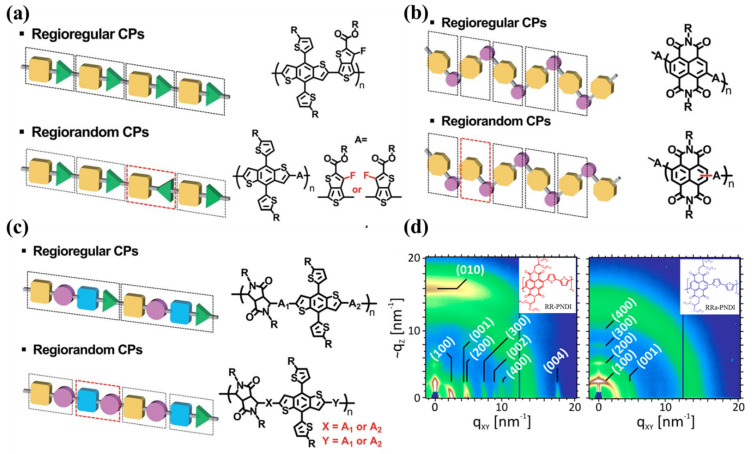
Schematic diagrams and structural representations of (**a**) directional, (**b**) positional, and (**c**) sequential RRs of D-A conjugated polymers that were adapted from reference [48] with permission from Copyright 2022 Royal Society of Chemistry. (**d**) Plots of 2D GIXD detector intensities for annealed PNDI films spin-coated from CB solutions that were adapted from reference [54] with permission from Copyright 2014 American Chemical Society.

It is axiomatic that the geometrical regularity of the molecular structure is critical for polymer crystallization [46,47]. For conjugated polymers, the regioregularity describes the geometrical symmetry of each monomer unit along the backbone. Kim et al., proposed that there are three types of RRs for D-A conjugated polymers: directional, positional and sequential as shown in Figure 2. The first RR was defined by the directional orientation of the functional groups, or side chains, along the backbone. The second RR was defined by the positional orientation of covalent bonds between monomer units in the backbone. The third RR was defined by the fraction of identical D-A sequences in D-A alternating copolymers [48]. Regardless of the type of RRs, regioregular (RRe) D-A conjugated polymers generally result in a better crystallinity than regiorandom (RRa) ones, especially along the π-π stacking direction [49,50,51,52,53]. For example, Neher et al., prepared two poly[[N,N′-bis(2-octyldodecyl)-1,4,5,8-naphthalenediimide-2,6-diyl]-alt-5,5′-(2,2′-bithiophene)]s (PNDIs) with an RRe and RRa backbone, respectively. The plots of the two-dimensional (2D) X-ray diffraction (GIXD) detector intensities of the RRe PNDI showed obvious diffraction peaks of lamellar stacking, π-π stacking and backbone stacking, suggesting a long-range ordered correlation of the crystallites in the film as shown in Figure 2d. However, only the weak diffraction peaks of lamellar stacking and backbone stacking were observed for the RRa PNDI, indicating a much poorer crystallinity [54]. The irregular backbone of RRa D-A conjugated polymers prevents the formation of ordered structure along the π-π stacking direction.

### 2.2. The Stiffness of the Backbone

The flexibility of polymer materials stems from the rotational motion of C-C bonds. If the rotational motion of C-C bonds is prevented, the polymers will show rigidity. For D-A conjugated polymers, the conjugated backbone and the steric hindrance of side chains hinder the rotational motion of C-C bonds, and, thus, the rigidity of D-A conjugated polymers is strong. Besides, it is believed that the rigid coplanar conformation of conjugated polymer backbones is beneficial to the intrachain and interchain charge transport [55,56,57]. Thus, chemical scientists tend to increase the stiffness of backbones when designing the chemical structure of D-A conjugated polymers [58]. The design strategies include noncovalent-interaction-locked conjugated polymers [59,60,61], double-bond-linked conjugated polymers [62,63] and conjugated ladder polymers [64,65]. As a result, the backbone stiffness of D-A conjugated polymers is much stronger than that of flexible polymers and p3ATs [66,67,68,69].

The backbone stiffness of polymers could be quantified by the persistence length (*l*_p_) which describes how long it takes for the backbone to bend 90 degrees on average [70]. The *l*_p_ value of D-A conjugated polymers is larger than 5 nm in general, while the *l*_p_ values of polyethylene (PE) and p3ATs are only about 0.7 nm and 2.8 nm, respectively [34,66]. The rigid backbone will affect the crystallization process and the crystal structure of D-A conjugated polymers as discussed in the following sections. For example, a typical feature is that D-A conjugated polymers generally adopt an extended chain in crystals [71], which is significantly different from the folded chain conformation in flexible polymers and p3ATs [72,73,74,75]. 

### 2.3. The Anisotropic Interchain Interactions

Due to the free rotation of C-C bonds, the intermolecular interactions in flexible polymers can be divided into two categories: parallel to the chain and perpendicular to the chain. In the plane perpendicular to the chain, flexible polymer chains could adopt a low-energy conformation during the crystallization process. As a result, a helical chain conformation is a reasonable choice in flexible polymer crystals [76]. For D-A conjugated polymers, the rotational motion of C-C bonds is prevented, and, thus, the interchain interaction is anisotropic. The intermolecular interactions in D-A conjugated polymers can be divided into three directions: the alkyl side chain, the π-π stacking and the backbone. Among them, the interchain interaction along the π-π stacking direction is much stronger than the other directions. In the presence of strong intermolecular interactions, conjugated small molecules and p3ATs tend to form one-dimensional fibril, or nanowire, structures with the length axis along the π-π stacking interaction [77,78]. However, the length direction of D-A conjugated polymer fibrils, or nanowires, is generally along the backbone direction [71,79]. The possible reasons for this were discussed in Section 3. In addition, the three directions above are orthogonal to each other according to the chemical structure of D-A conjugated polymers. This could be the reason for typical orthorhombic crystal unit cells being found in mainly D-A conjugated polymer crystals [80,81,82,83]. In general, the (h00), (0j0) and (00k) diffraction peaks are regarded as the stacking of D-A conjugated polymer chains along the alkyl side chain (the lamellar stacking), the π-π stacking and the backbone directions, respectively.

### 2.4. The Role of Alkyl Side Chains

**Figure 3:**
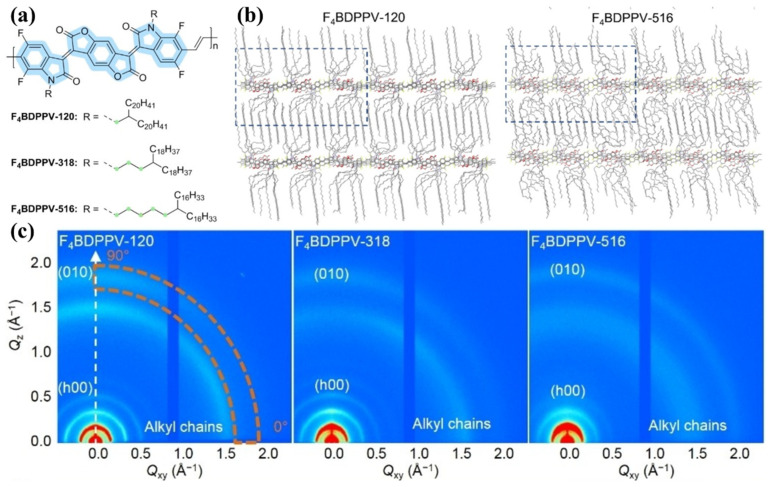
(**a**) Chemical structures of the three F_4_BDPPV polymers. (**b**) Equilibrated snapshots of packing structures extracted from MD simulations for F_4_BDPPV-120 and F_4_BDPPV-516. (**c**) 2D GIWAXS patterns of the three F_4_BDPPV polymers. These were adapted from reference [86] with permission from Copyright 2022 Wiley.

The alkyl side chains may affect the crystallization of D-A conjugated polymers in two ways. On the one hand, the alkyl side chains may also crystallize if their length is long enough [84,85]. For example, Pei et al., designed three tetrafluorinated benzodifurandione-based poly(p-phenylene vinylene)s (F4BDPPVs) with different branched alkyl chains. The distance of the branching point from the backbone was two, four and six single bonds as shown in Figure 3a. For all of the three polymers, alkyl side chain crystallization with diffraction peaks around 1.5 Å^−1^ was observed in the 2D wide-angle X-ray scattering (GIWAXS) patterns as shown in Figure 3c. In addition, the crystallinity of alkyl side chains was weaker when the branching point was further away from the backbone, which was consistent with the results of molecular dynamics (MD) simulations as shown in Figure 3b [86]. On the other hand, the alkyl side chains may affect the crystallinity of backbones. Long alkyl side chains generally increase the distance of lamellar stacking, but they have limited influence on the distance of π-π stacking [87,88]. The flexibility of side chains appears to be an important factor in influencing the distance of π-π stacking. Wang et al., proposed that the distance of π-π stacking could be decreased by using more flexible oligo(ethylene glycol) (OEG) side chains [89,90]. Pei et al., proposed that increasing the distance of the branching point from the backbone is a powerful strategy for decreasing the distance of π-π stacking [86,91,92]. The possible reason for this is that further branching point positions could increase the flexibility of side chains, and, thus, the side chains could adopt a metastable conformation in narrower spaces.

Besides the above features, factors such as the solubility, molecular weight (MW) and molecular weight distribution of D-A conjugated polymers also affect the crystallization of D-A conjugated polymers. These factors are common characteristics of polymer materials, and we dispersedly discussed them in the following sections.

## 3. The Crystallization Process of D-A Conjugated Polymers

The formation of crystals may be governed by thermodynamics or kinetics. In the thermodynamic equilibrium state, the length of the crystal in three directions could be expressed as [71,77]

(1) $$\frac{L_{1}}{2\sigma_{1}+\varepsilon_{1}}=\frac{L_{2}}{2\sigma_{2}+\varepsilon_{2}}=\frac{L_{3}}{2\sigma_{3}+\varepsilon_{3}}$$

where *L*_i_ is the length of the crystal along the *i* direction; *σ*_i_ is the interfacial energy per unit area of surfaces perpendicular to the *I* direction; and *ε*_i_ is the interaction energies per unit area between two adjacent molecules perpendicular to the *i* direction (*i* = 1, 2, 3). For flexibility polymers, there is a competition between intramolecular nucleation (resulting in folded chain conformation) and intermolecular nucleation (resulting in extended chain conformation). The energy barrier for intramolecular nucleation is smaller than that for intermolecular nucleation, and, thus, intramolecular nucleation is faster than intermolecular nucleation. If the polymer chains are long and flexible enough, intramolecular nucleation will dominate and will result in lamellar crystals. The folded chain conformation in lamellar crystals is unstable and will slowly unfold to approach the more stable extended chain conformation in the equilibrium crystals. On the contrary, intermolecular nucleation is dominating when polymer chains are short and rigid. The nucleation process often passes through a transient mesophase with looser molecular packing to lower the high nucleation barrier [72]. In the case of D-A conjugated polymers, intermolecular nucleation is dominating because they adopt an extended chain conformation in crystals. However, crystallization kinetics seems to play an important role in the crystallization process, and the resulting crystals are not thermodynamically stable in general. One reason for this is that the length direction of D-A conjugated polymer crystals is generally along the backbone [71]. If the crystallization process is governed by thermodynamics, according to Equation (1), the length direction of D-A conjugated polymer crystals will be along the π-π stacking direction due to the strong intermolecular π-π stacking interactions. Another reason is that the crystal of higher MW D-A conjugated polymers will be slender if the crystallization process is governed by thermodynamics, which is against the experimental results of Choi et al. [80]. Therefore, understanding the crystallization kinetics of D-A conjugated polymers is critical. Because the crystallization of D-A conjugated polymers begins in the solution, the chain conformation in the solution as well as the nucleation and growth processes are key factors affecting the crystallization process as shown in Figure 1e–g.

### 3.1. The Chain Conformation in the Solution

In the initial solution, polymer chains may form unimer conformations (coil and rod) or aggregates (short-range, ordered and amorphous). Amorphous aggregates are hard to disentangle, and they will maintain their morphologies during the crystallization process [93,94]. Polymer chains with unimer conformations can reach the growth front of small crystals to form larger crystals, and, thus, they are essential for the crystallization process. To achieve unimer conformations in solutions, a “good” solvent should be used to dissolve the D-A conjugated polymers. The “good” solvent should not only show a high solubility of the D-A conjugated polymer but also should have the ability to prevent the disordered aggregate of the polymer chains [95]. Short-range ordered aggregates in solutions are also important to the crystallization process because they can act as nuclei [96]. In general, heterogeneous nucleation happens easier than homogeneous nucleation because the former needs to overcome less Gibbs free energy compared to the latter. However, if the initial solution contains too many ordered aggregates, the resulting crystal size will be very small due to the absence of unimer chains. Therefore, the proper amount of short-range ordered aggregates is beneficial to the crystallization process.

**Figure 4:**
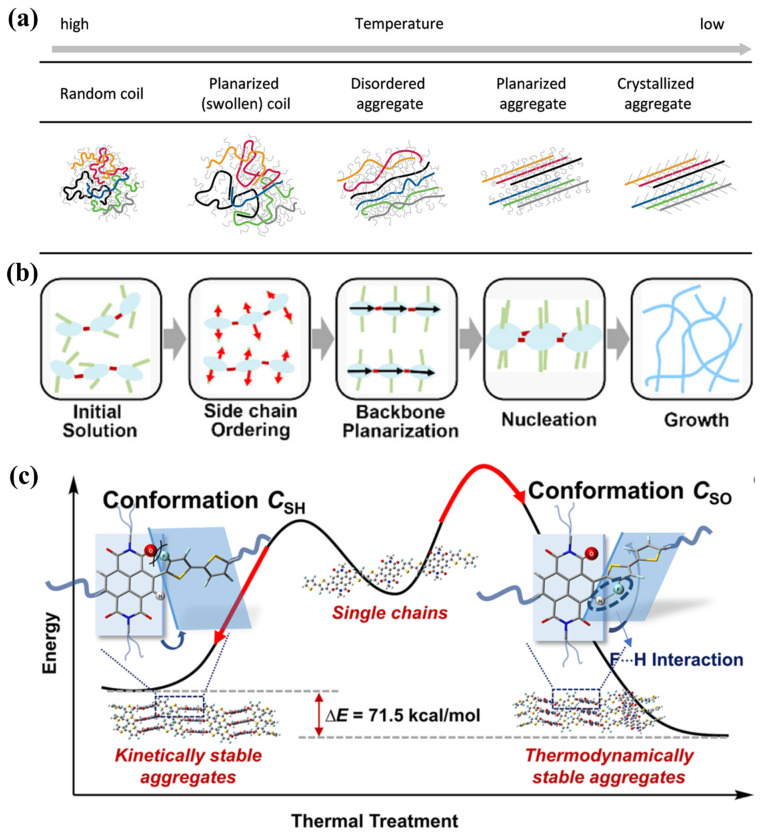
(**a**) Schematic diagram of the evolution of conjugated polymer chain conformations in solution during the cooling process, which was adapted from reference [104] with permission from Copyright 2017 American Chemical Society. (**b**) Schematic diagram of the evolution of PNDI chain conformations in the BN solution during the aging process, which was adapted from reference [69] with permission from Copyright 2021 American Chemical Society. (**c**) Schematic diagram of the potential energy and the corresponding chain conformations of PNDI-4F2T before and after thermal treatment, which was adapted from reference [106] with permission from Copyright 2021 American Chemical Society.

The chain conformation of D-A conjugated polymers could be adjusted by changing the temperature [97,98,99,100], changing the solvent [101] and adding solvent additives [102,103]. Koöhler et al., analyzed the aggregate forms of three types of conjugated materials (homopolymers, D-A type polymers and low-MW compounds) in solution during the cooling process. They proposed that the conformation of molecular chains in solution evolved from a random coil to a planarized coil, a disordered aggregate, a planarized aggregate and a crystallized aggregate gradually as shown in Figure 4a. The backbones were supposed to form an ordered structure prior to the side chins through a series of processes, including backbone planarization, order–disorder collapse and then backbone planarization [104]. However, the side chains may form an ordered structure prior to the backbone [105]. For example, Han et al., prepared PNDI nanowires by first aging the PNDI solution in 1-bromonaphthalene (BN) for 4 days and then dropcasting it onto the glass substrates. They proposed that the side chain ordering driven by the unfavorable BN-side-chain interactions was prior to the backbone planarization as shown in Figure 4b [69].

In addition to the planarization and aggregation of D-A conjugated polymer chains, D-A conjugated polymers may also form different backbone conformations due to the intrachain interactions. An example was provided by Pei et al., as shown in Figure 4c [106]. They used the fluorine atom to replace the hydrogen atom in the thiophene units of PNDI, and the new polymer was named PNDI-4F2T. There are two types of backbone conformations for the PNDI family: C_SO_ and C_SH_. In PNDT, they found that the energies of the two conformations were almost identical. However, due to the formation of additional fluorine–hydrogen (F···H) interactions, the energy of C_SO_ was much lower than that of C_SH_ in PNDI-4F2T. Interestingly, they showed that the conformation C_SH_ is kinetically stable and could change to the thermodynamically stable conformation C_SO_ upon thermal treatment [106].

### 3.2. The Nucleation Process

It seems easy to nucleate in D-A conjugated polymers because of their rigid backbones and strong interchain π-π stacking interactions. One can guess that thermal-induced local planarized backbones may meet each other through chain collapse or thermal motion and then form a short-range ordered structure due to the π-π stacking interactions. According to the classical nucleation theory, the nucleus can exist stably in the solution only when its size is larger than the critical value [107,108]. For P3HT, Xu et al., proposed that the critical nucleus contained about 30 crystalline units in weakly supersaturated solutions. They also found that the number of crystalline units within the critical nucleus was independent from the degree of supersaturation, which was against the predictions of the classical nucleation theory [109]. However, calculating the critical size of the D-A conjugated polymer nucleus is still a challenge. Using small-angle X-ray scattering (SAXS), Toney et al., proposed that the solution of a diketopyrrolopyrrole-based (or DPP-based) polymer (PDPP) contained short-range ordered aggregates with a size of about 25 nm along the backbone direction when the solvent addition 1-chloronaphthalene (CN) was used [102]. Therefore, in their case, the critical size of the nucleus should be less than 25 nm.

**Figure 5:**
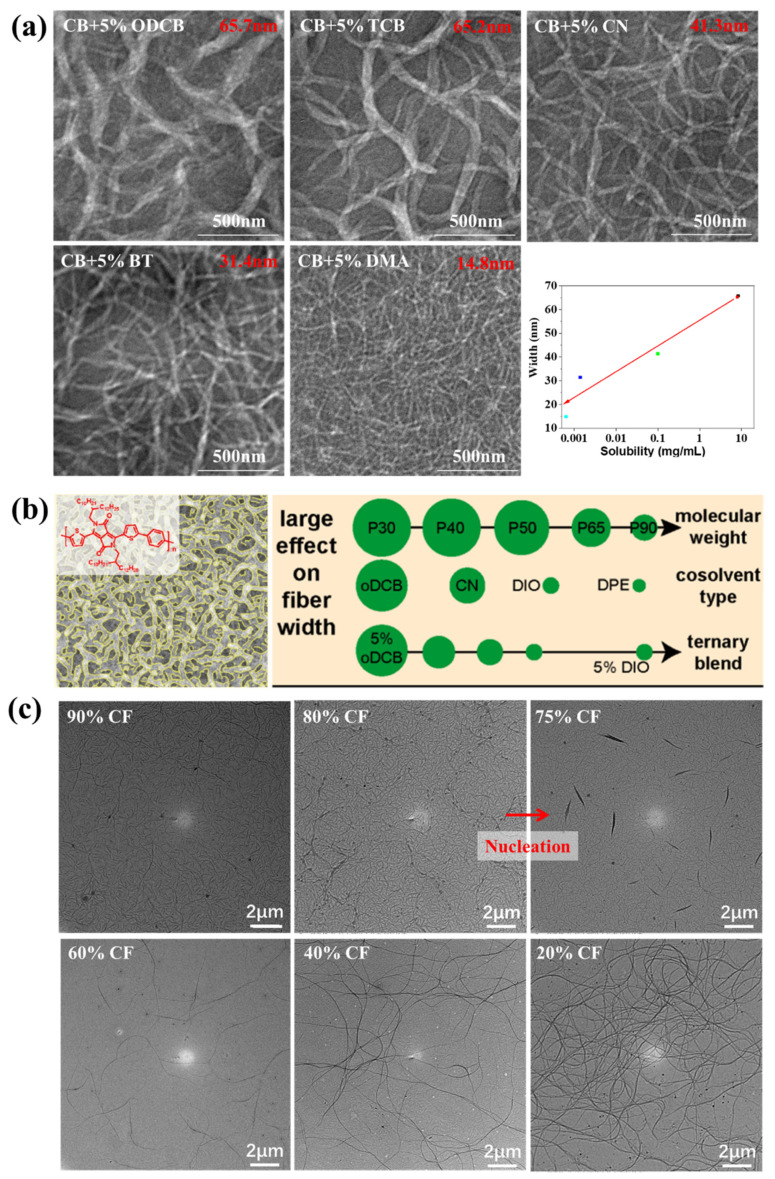
(**a**) Transmission electron microscopy (TEM) images of PDBT:PC_71_BM blend films processed with different solvent additives and the effect of the solubility of PDBT in solvent additives on the fibril width of PDBT, which were adapted from reference [96] with permission from Copyright 2015 Elsevier B.V. (**b**) Factors with a large effect on the fibril width of PDPPTPT in the PDPPTPT:PC_71_BM blend films, which were adapted from reference [111] with permission from Copyright 2021 American Chemical Society. (**c**) TEM images of PDBT nanowires during the slow evaporation process, which were adapted from reference [112] with permission from Copyright 2021 Elsevier Ltd.

The nucleation process may be homogeneous or heterogeneous. As we mentioned in Section 3.1, D-A conjugated polymer chains may form short-range ordered aggregates with the proper temperature and solvent. The formation of these short-range ordered aggregates could be regarded as a self-seeding nucleation process which is a special case of heterogeneous nucleation. Extra nucleating agents which could promote the heterogeneous nucleation process are rarely used in conjugated polymers probably because they may reduce the electronic performance of conjugated polymers by introducing electronic traps. For conjugated polymers, heterogeneous nucleation generally occurs on the surface of the substrate during the film formation process or in the solution during self-seeding. In the former situation, the surface energy of the substrate is critical for heterogeneous nucleation. For example, Diao et al., proposed that decreasing the surface energy could reduce the free energy barrier of heterogeneous nucleation and, thus, resulted in increased crystallinity and an increased degree of molecular ordering [110]. Self-seeding nucleation is an effective strategy for controlling the crystallinity of conjugated polymers. Pei et al., controlled the solution-state aggregation of a D-A conjugated polymer by changing the temperature, and the results indicated that the polymer film deposited at a medial temperature showed the highest crystallinity [99]. Han et al., controlled the fibril width of 3,6-bis-(thiophen-2-yl)-N,N′-bis(2-octyl-1-dodecyl)-1,4-dioxo-pyrrolo[3,4-c]pyrrole and thieno[3,2-b]thiophene copolymer (PDBT) in PDBT: [6,6]-phenyl-C_71_-butyric acid methyl ester (PC_71_BM) blend films by adjusting the solubility of PDBT in solvent additives. They found that a lower solubility resulted in a narrower fibril width as shown in Figure 5a. They proposed that solvent additives could promote the nucleation of PDBT in solution. Increasing the amount of solvent additives or decreasing the solubility of PDBT in solvent additives resulted in more nuclei, and, thus, the width of fibrils decreased [96]. Later, Janssen et al., studied the effect of many factors on the fibril width of an alternating copolymer of diketopyrrolopyrrole and a thiophene-phenyl-thiophene oligomer (PDPPTPT) in blend films. They concluded that factors such as MW, cosolvent type and cosolvent blend had a large effect on the fibril width of PDPPTPT as shown in Figure 5b. They agreed with Han et al.’s conclusion that the solubility of PDBT in solvent additives determined the fibril width because the change of the above three factors also induced the decrease of solubility. However, they proposed that the mechanism of nucleation was homogeneous nucleation because the amount of solvent had a limited effect on the fibril width in their study. Also, they thought that the actual nucleation mechanism was a combination of homogeneous and heterogeneous nucleation [111].

Homogeneous nucleation generally occurs when the crystallization process begins from a homogeneous solution. In fact, it is difficult to distinguish between homogeneous nucleation and self-seeding nucleation because the chain conformation is complex, and the critical size of the nucleus is unknown. Han et al., prepared PDBT nanowires by slowly evaporating the solution. The solvent was composed of a low-boiling good solvent chloroform (CF) and a high-boiling marginal solvent o-dichlorobenzene (ODCB). During the evaporation process, PDBT crystals appeared and then gradually grew into long nanowires as shown in Figure 5c. They found that decreasing the evaporation speed of CF was critical for the formation of nanowires. A fast evaporation rate resulted in the amorphous aggregates of PDBT. Therefore, they thought that the nuclei were formed during the evaporation process through a homogeneous nucleation mechanism.

### 3.3. The Growth Process

**Figure 6:**
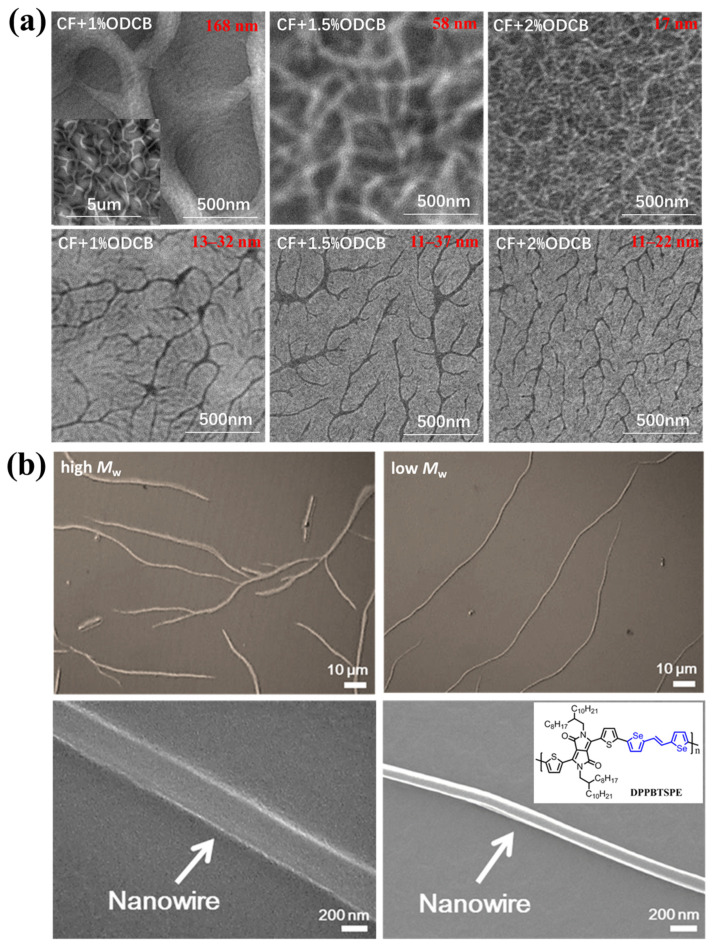
(**a**) TEM images of PDQT:PC_71_BM blend films (**top**) and pure PDQT films (**bottom**) processed with different amounts of solvent additive ODCB, which were adapted from reference [113] with permission from Copyright 2017 Wiley Periodicals LLC. (**b**) TEM images of high- and low-MW DPPBTSPE nanowires, which were adapted from reference [80] with permission from Copyright 2015 American Chemical Society.

The growth direction of D-A conjugated polymer crystals may be along the alkyl side chain, the π-π stacking and the backbone. Understanding which direction is dominant during the growth process is also very important. For example, the length direction of D-A conjugated polymer crystals is along the backbone in most cases [71]. Han et al. [96] and Janssen et al. [111] proposed that the fibril width of D-A conjugated polymers depends on the nucleation density, indicating that the fibrils could grow along the π-π stacking direction (the chains adopted edge-on orientation). However, the growth of nanowires is mainly along the backbone direction, as shown in Figure 5c. To further understand the growth direction of D-A conjugated polymer crystals, our group studied the effect of nucleation density on the morphologies of poly[2,5-bis(2-octyldodecyl)pyrrolo-[3,4-*c*]pyrrole-1,4(2H,5H)-dione-*alt*-2,2′: 5′,2″: 5″,2‴-quaterthiophene] (PDQT) pure films and blend films as shown in Figure 6a. We found that increasing the amount of the solvent additive ODCB resulted in a narrower fibril width in blend films, which was consistent with the results of Han et al. [96]. In the case of pure films, the crystal density increased when the amount of ODCB increased, but the fibril width had a limited change. The results indicated that the dominant growth direction of PDQT crystals was different in blend films and pure films. We proposed that the growth of PDQT fibrils was in a confined space for blend films due to the presence of PC_71_BM, as the fibrils could not grow along the length direction when the crystal size in this direction was large enough, and, thus, the fibrils could only grow along the radial direction [113]. Another example is the effect of MW on the crystal sizes of D-A conjugated polymers. Choi et al., synthesized a highly crystalline DPP-based polymer (DPPBTSPE). They found that the aspect ratio of nanowires for low-MW (8 kDa) DPPBTSPE was much higher that of high-MW (68 kDa) DPPBTSPE during the slow crystallization process in the dilute solution as shown in Figure 6b [80]. This result could not be explained by Equation (1). Additionally, if MW only affects the nucleation process, the aspect ratio of nanowires for high-MW and low-MW DPPBTSPE should be similar. Therefore, the MW must also affect the growth process of D-A conjugated polymer crystals.

In fact, understanding the growth process of D-A conjugated polymers is a challenge. This is because most polymer crystallization theories were based on the hypothesis of folded chains [72,73,74,75,114,115], which is different from the extended chain conformation in crystals for D-A conjugated polymers [71]. Think about the nature of the crystallization process; it is actually the motion of molecules. The movement types of molecules could be divided into translational motion and rotational motion, which could be realized by the diffusion and the conformational transition of molecules during the crystallization process, respectively. For small molecules, their conformational transition process is very fast because of their small molecular sizes. The growth theory of small-molecule crystals is mainly concerned about the diffusion process [116]. In the case of flexible polymers, the conformational transition process is rather important because of the rich chain conformations. The fold of polymer chains during the growth process could be regarded as the conformational transition process. D-A conjugated polymers have the feature of rigidity because of the conjugated backbone and the feature of flexibility because of the long chains, and, thus, both the diffusion process and the conformational transition process are important during the growth process. Based on the above analyses, our group proposed a crystallization theory based on diffusion and conformational transition (D-CT) to quantitatively explain the crystallization process of D-A conjugated polymers [117].

**Figure 7:**
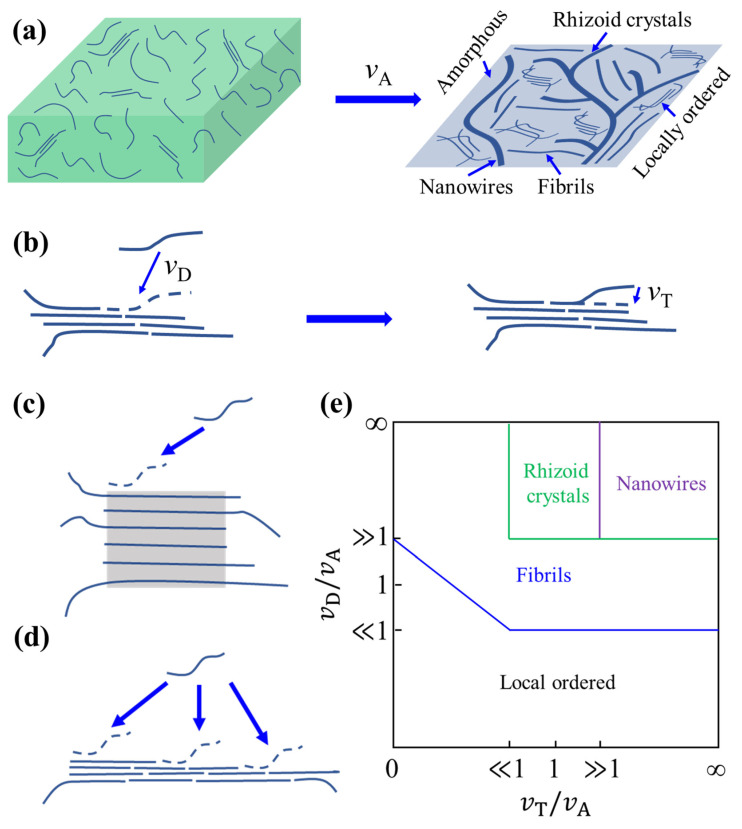
(**a**–**d**) The schematic diagrams of the crystallization process of D-A conjugated polymer crystals based on the D-CT theory. (**a**) The aggregate process of polymer chains. (**b**) The diffusion and conformational transition processes of polymer chains. (**c**,**d**) The possible growth directions of a single polymer chain. (**e**) The diagram of how *v*_D_, *v*_A_ and *v*_T_ values affect the morphology of D-A conjugated polymer crystals. These were adapted from reference [117] with permission from Copyright 2022 Elsevier Ltd.

In consideration of the fact that some polymer chains may form amorphous aggregates during the crystallization process, we used the average aggregate rate (*v*_A_) to replace the growth rate of crystals. The *v*_A_ was proposed to describe how many polymer chains attach to every aggregate per unit time as shown in Figure 7a. According to this definition, *v*_A_ could be expressed as [117]

(2) $$v_{A}=\frac{\left(c_{0}V_{0}-c_{e}V_{e}\right)}{\rho_{N}V_{0}t_{c}}$$

where *c*_0_ and *c*_e_ are the solution concentrations at the beginning and the end of the crystallization process, respectively. *V*_0_ and *V*_e_ are the solution volumes at the beginning and at the end of the crystallization process, respectively. $\rho_{N}$ is the density of the nuclei, and *t*_c_ is the crystallization time. The key hypothesis of the D-CT theory was that the growth of D-A conjugated polymer crystals involved two steps: (a) the chain segments diffused to the growth front of the crystals from the solution and (b) the rest part of the polymer chain transformed into the extended chain conformation as shown in Figure 7b [117]. The first process was named “diffusion”, and the average diffusion rate (*v*_D_) was proposed to describe how many polymer chain segments could reach the growth front of every crystal per unit time. According to this definition, *v*_D_ could be expressed as [117]

(3) $$v_{D}=\frac{kTn_{s}c_{t}}{\zeta l_{D}}N_{f}S_{f}\;P\left(S\right)$$

where *k* is the Boltzmann constant, *T* is temperature, *n*_s_ is the average number of chain segments in every polymer chain, *c*_t_ is the solution concentration, *ζ* is the friction coefficient, *l*_D_ is the average diffusion distance of the chain segment, *N*_f_ is the number of growth fronts in every crystal, *S*_f_ is the effective surface area of every growth front and *P*(S) is the probability that the conformation of the chain segment matches with the growth front. The second process was named “conformational transition,” and the average conformational transition rate (*v*_T_) was proposed to describe how many polymer chains could transform into the extended chain conformation along the growth front of every crystal per unit time. According to this definition, *v*_T_ could be expressed as [117]

(4) $$v_{T}=\frac{N_{f}}{An_{s}}e^{-\frac{\Delta G_{T}}{kT}}$$

where *A* is a constant and Δ*G*_T_ is the energy difference during the conformational transition process of the chain segment.

Another important hypothesis of the D-CT theory was that the growth rate of more thermodynamically stable D-A conjugated polymer crystals along the backbone direction (*v*_b_) should not be faster than those of the π-π stacking direction (*v*_π-π_) [117]. That is, the crystal structure shown in Figure 7d is more stable than that shown in Figure 7c. According to this hypothesis,

(5) $$\frac{v_{b}}{v_{\pi -\pi }}=\frac{N_{b}}{N_{\pi -\pi }}=\frac{l_{b}/L_{e}}{l_{\pi -\pi }/d_{\pi -\pi }}\geq 1$$

where *N*_b_ and *N*_π-π_ are the average number of polymer chains along the backbone and the π-π stacking directions, respectively; *l*_b_ is the size of the crystal along the backbone direction; *l*_π-π_ is the size of crystal along the π-π stacking direction; *L*_e_ is the average length of the extended chain; and *d*_π-π_ is the π-π spacing. Equation (5) could be rewritten as

(6) $$\frac{l_{b}}{l_{\pi -\pi }}\geq \frac{L_{e}}{d_{\pi -\pi }}$$

By analyzing how *v*_D_, *v*_A_ and *v*_T_ values affected the morphology of D-A conjugated polymer crystals, the D-CT theory could be used to qualitatively predict the crystal structure of D-A conjugated polymers as shown in Figure 7e [117].

The D-CT theory could be used to explain the above experimental results associated with the growth process. For example, the *L*_e_ value of D-A conjugated polymers is generally larger than dozens of nanometers (based on the MW), and the *d*_π-π_ value is about 0.3–0.4 nm. According to Equation (6), the *l*_b_/*l*_π-π_ value of D-A conjugated polymer crystals is generally larger than 100. This could explain why the length direction of D-A conjugated polymer crystals is along the backbone [71,117]. Additionally, decreasing the MW results in a higher *v*_D_ value, and, thus, polymer chains reach the crystal growth front along the backbone direction easier. As a result, the crystals of the low-MW polymer are closer to the thermodynamically stable structure (high aspect ratio) [117].

## 4. The Crystal Structure

Once a three-dimensional ordered structure is formed through the nucleation and growth process, it can be regarded as a crystal. As mentioned above, D-A conjugated polymer crystals are generally orthorhombic crystals because the intermolecular interactions along the alkyl side chain as well as the π-π stacking and the backbone directions are orthogonal to each other. In consideration of the fact that D-A conjugated polymer thin films, or blend films, are two-dimensional structures, the molecular orientation of D-A conjugated polymers in crystals is an important factor affecting their applications [118,119]. Another factor associated with the crystal structure is polymorphism [120]. We will discuss them in this section.

### 4.1. The Molecular Orientation

According to which crystal axis is perpendicular to the substrate, the molecular orientation of conjugated polymers could be divided into three types: face-on, edge-on and flat-on. In films, conjugated polymers generally adopt a face-on or edge-on orientation [121,122]. If the π-π stacking direction is perpendicular to the substrate, the molecular orientation is named “face-on”. On the other hand, the molecular orientation is named “edge-on” if the side chain stacking direction is perpendicular to the substrate. In particular cases [123,124], conjugated polymers may adopt a “flat-on” orientation where the backbone is perpendicular to the substrate. Additionally, the backbones may form an ordered arrangement in the direction parallel to the substrate if an appropriate shear force is applied during the film formation process [125,126,127,128]. This situation will not be discussed here because the resulting structure belongs to film structure or texture structure.

**Figure 8:**
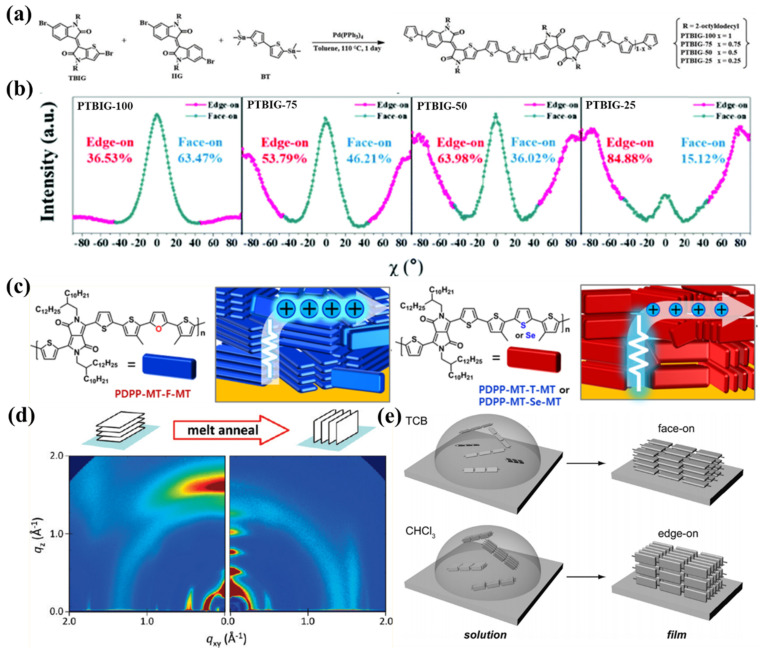
(**a**) Synthetic scheme of the four polymers. (**b**) The relative content of the edge-on and face-on orientations of the four polymers calculated from the results of 2D-GIXD, which was adapted from reference [129] with permission from Copyright 2020 Royal Society of Chemistry. (**c**) Schematic diagrams of the chemical structure and the molecular orientation of the three PDPPs, which were adapted from reference [130] with permission from Copyright 2022 American Chemical Society. (**d**) Schematic diagrams of the molecular orientation of PNDI, which were adapted from reference [131] with permission from Copyright 2011 American Chemical Society. (**e**) Schematic diagrams of the surface molecular orientation in solutions and films, which were adapted from reference [133] with permission from Copyright 2016 Wiley.

During the film formation process, the D-A conjugated polymers will adopt a random orientation if the nucleation process is homogeneous. The orientation of D-A conjugated polymers may be formed through three approaches. The first approach is heterogeneous nucleation on the surface of the substrate. In this situation, the conjugated polymer chains adopt an appropriate orientation to increase the interactions between the substrate and the conjugated polymers. Adjusting the molecular structure of D-A conjugated polymers can influence the interactions between the substrate and the conjugated polymers, and, thus, the thermodynamically stable orientation of D-A conjugated polymers may also change. For example, Yang et al., synthetized four brand-new thieno-benzo-isoindigo-based (or TBIG-based) copolymers by changing the ratio of TBIG and the isoindigo (IIG) units as shown in Figure 8a. The backbone coplanarity increased when the copolymer contained more TBIG units. The 2D-GIXD results indicated that the relative content of the edge-on orientation increased when the coplanarity of the backbone increased as shown in Figure 8b [129]. In another example, Hwang et al., synthetized three PDPPs by using furan and selenophene units to replace the thiophene unit of the backbone. The results indicated that the PDPP containing furan units adopted a face-on orientation, while the PDPP containing thiophene or selenophene units adopted an edge-on orientation as shown in Figure 8c [130].

The second approach is the orientation of aggregates when D-A conjugated polymers are preaggregated in the solution. For example, PDNI mostly adopted a face-on orientation in spin-coated films. When the films underwent a melt-annealed process, PNDI changed to the edge-on orientation as shown in Figure 8d [131]. The edge-on orientation resulted from the heterogeneous nucleation happening on the surface of the substrate, while the face-on orientation could be attributed to the chain collapse of the aggregates formed via the coiling of individual polymer chains [101,132]. In another example, Pisula et al., found that difluorobenzothiadiazole-based (or FBT-based) polymers (FBT-Th_4_(1,4)) adopted an edge-on orientation in the film casted from CF, but the orientation changed to face-on when 1,2,4-trichlorobenzene (TCB) was added into the solution. They proposed that FBT-Th_4_(1,4) preaggregated in CF solution due to strong intermolecular π-π stacking interactions, and the aggregates lay flat on the substrate (face-on) to maximize the interaction between the polymer and substrate. On the contrary, TCB efficiently lowered the aggregates in the solution and, thus, resulted in a face-on orientation [133]. Therefore, competition between the orientation of aggregates formed in the solution and heterogeneous nucleation happening on the surface of the substrate may be the reason for different molecular orientations.

The last approach is the orientation of D-A conjugated polymer crystals. During the film formation process, the crystals grown in solution will adopt an appropriate orientation to maximize the interaction between the crystal and substrate. The evidence for this is that conjugated polymer nanowires prepared in the solution generally adopted edge-on orientations when they were cast on the substrate [80,82,112,134,135].

### 4.2. Polymorphism

**Figure 9:**
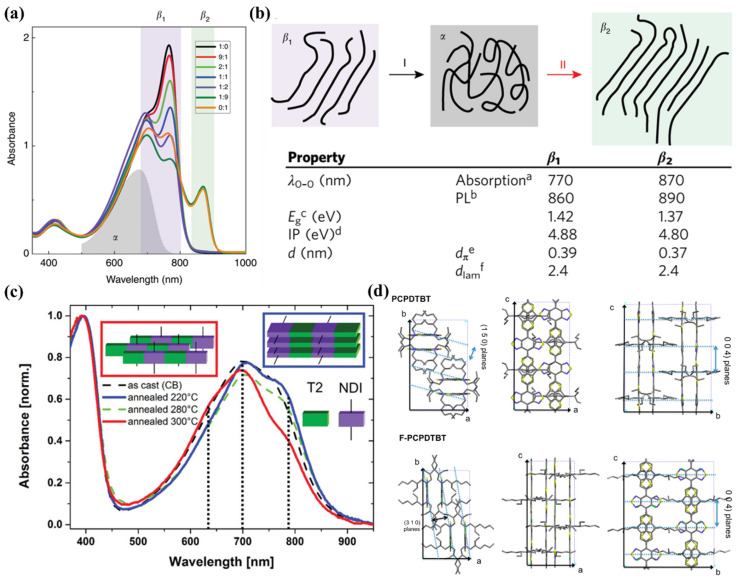
(**a**) UV-vis spectra of D-PDPP4T-HD dissolved in CF and TCB mixtures. (**b**) Schematic illustration and properties of the two polymorphs, which were adapted from reference [136] with permission from Copyright 2019 Nature Publishing Group. (**c**) UV-vis spectra of PNDI films melt-annealed at different temperatures, which were adapted from reference [137] with permission from Copyright 2014 Wiley. (**d**) The structural models of PCPDTBT and F-PCPDTBT crystals, which were adapted from reference [139] with permission from Copyright 2015 American Chemical Society.

The polymorphism of D-A conjugated polymers has been reported by several groups. Janssen et al., found that the solution of D-PDPP4T-HD (a PDPP) in the CF and TCB mix solvent contained two semi-crystalline polymorphs (*β*_1_ and *β*_2_). When the content of TCE increased, the *β*_1_ phase transformed into the amorphous α phase, and then a new *β*_2_ phase appeared as shown in Figure 9a,b. The two polymorphs had distinctly different parameters such as the maximal absorption peak, the optical bandgap and the π-π stacking distance [136]. PNDI also exhibited two distinct polymorphs when melt-annealed at different temperatures [137,138]. Brinkmann et al., proposed that PNDI adopted a face-on orientation with the acceptor and donor units stacked in segregated columns when melt-annealed at 220 °C as shown in Figure 9c. However, PNDI changed to an edge-on orientation with a mixed stacking of the acceptor and donor units when melt-annealed at 300 °C [137]. Changing the chemical structure may also result in new polymorphs [139,140]. For example, Brinkmann et al., proposed that a new polymorph was achieved when using a F atom to replace the H atom in the benzothiadiazole unit of poly[2,6-(4,4-bis(2-ethylhexyl)-4H-cyclopenta[2,1-b;3,4-b′]dithiophene)-alt-4,7(2,1,3-benzothiadiazole)] (PCPDTBT) as shown in Figure 9d [139]. Although polymorphisms of several D-A conjugated polymers have been reported, the reason for these polymorphisms is not yet very clear. The formation of polymorphs could be related to the nucleation process because the crystal structure seems to be clear when the nuclei have formed.

## 5. The Crystal Morphology

When the growth process ends, the crystal morphology of D-A conjugated polymers in the film is then clear. According to size and shape, our group divided the crystal morphology into four types [117]. The first type was the locally ordered structures, and the *l*_b_ value were less than *L*_e_. If the *l*_b_ value was larger than *L*_e_ and less than several microns, they were called fibrils. On the other hand, the crystals were called nanowires if the *l*_b_ value was larger than several microns. In some cases, the fibrils, or nanowires, were branched, and they were called rhizoid crystals, as shown in Figure 6. We discussed them in this section.

### 5.1. Locally Ordered Structures

During the crystallization process, locally ordered structures may form in three situations. In the first case, the nucleation rate is very fast, and there are too many nuclei in the solution. The growth process is suppressed, and, thus, the *l*_b_ value is very small. In the second case, the growth rate is very fast, and the polymer chains do not have enough time to diffuse to the more stable growth front. The metastable crystals mainly grow along the π-π stacking direction due to the strong intermolecular π-π interactions. The *l*_π-π_ value could reach dozens of nanometers or even a few hundred nanometers, and this kind of structure is also called lamellar or fibrillar morphology [141,142,143,144]. In the third case, the nuclei or aggregates are surrounded by amorphous aggregates, and, thus, the growth of the crystals is hard. For example, the collapse process of a stiff polymer can result in an inner ordered structure [101,104], but the further growth of such an ordered structure along the backbone direction should be difficult.

**Figure 10:**
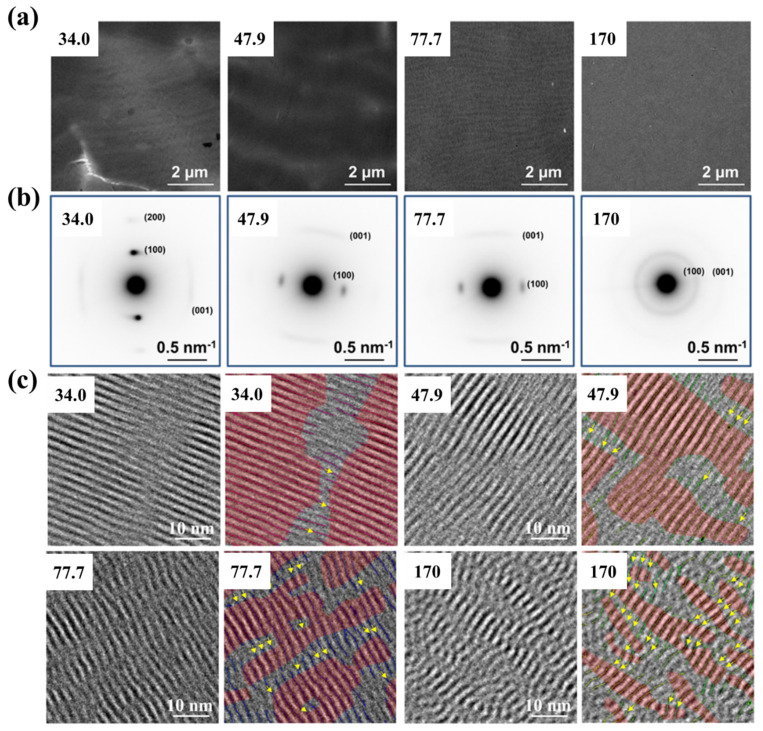
TEM images (**a**), SAED patterns (**b**) and HRTEM images (**c**) of drop-casted PNDI films with various MWs (kDa), which were adapted from reference [141] with permission from Copyright 2021 American Chemical Society.

The locally ordered structures can be directly observed using high-resolution transmission electron microscopy (HRTEM). Han et al., studied the effect of MW on the morphology of drop-casted PNDI films as shown in Figure 10. The TEM images showed an irregular lamellar morphology or homogeneous morphology, but the selected-area electron diffraction (SAED) patterns indicated that all of the films contained ordered structures. These ordered structures could be observed from the HRTEM images. The crystal size in the backbone direction was larger when the MW of PNDI was lower [141]. A lower MW resulted in larger *v*_D_ and *v*_T_ values; thus, the polymer chains could diffuse easier to the more stable growth front and accomplished the conformational transition process [117]. This could be the reason for the larger crystal size in the backbone direction.

### 5.2. Fibrils

**Figure 11:**
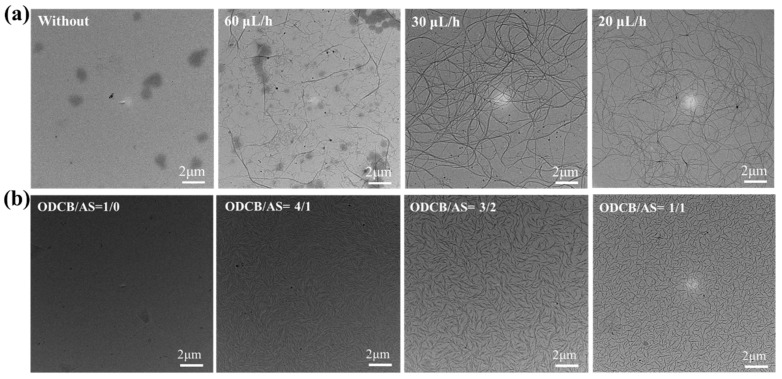
(**a**) TEM images of PDBT films dropcasted from slow evaporated solutions with different evaporation speeds, which were adapted from reference [112] with permission from Copyright 2017 Elsevier Ltd. (**b**) TEM images of PDPP films dropcasted from slow evaporated solutions with different cosolvents, which were adapted from reference [95] with permission from Copyright 2018 Wiley.

Fibrils are generally used to describe crystals with a high aspect ratio. The thickness of conjugated polymer films, or blend films, is around 100 nm, so the crystal size in the height direction is generally no more than dozens of nanometers. Fibril is a reasonable name for these slender crystals observed in the TEM images. If the length of the crystals is very long, the more relevant name nanowire is usually used. The difference between the locally ordered structure, fibril and nanowire is whether the extended chains can arrange freely in the crystals. Decreasing the *v*_A_ value is beneficial for the diffusion process and the conformational transition. The extended chains could then be arranged easier in the crystals, and, thus, the crystal morphology could be adjusted. For example, Han et al., studied the effect of evaporation speed on the crystal morphology of PDBT as shown in Figure 11a. Decreasing the evaporation speed increased the crystallization time and resulted in a lower *v*_A_ value. They found that the film morphologies were fibrils or locally ordered structures when the evaporation speed was fast, while they were nanowires when the evaporation speed was slow [112]. The size of fibrils could be controlled by adjusting the density of the nuclei. For example, Han et al., used the mixed solvent of ODCB and anisole (AS) as the cosolvent and studied the effect of the cosolvent on the crystal morphology of PDPP. They found that the fibril density decreased and the fibril size increased when the ratio of AS increased as shown in Figure 11a [95].

### 5.3. Nanowires

**Figure 12:**
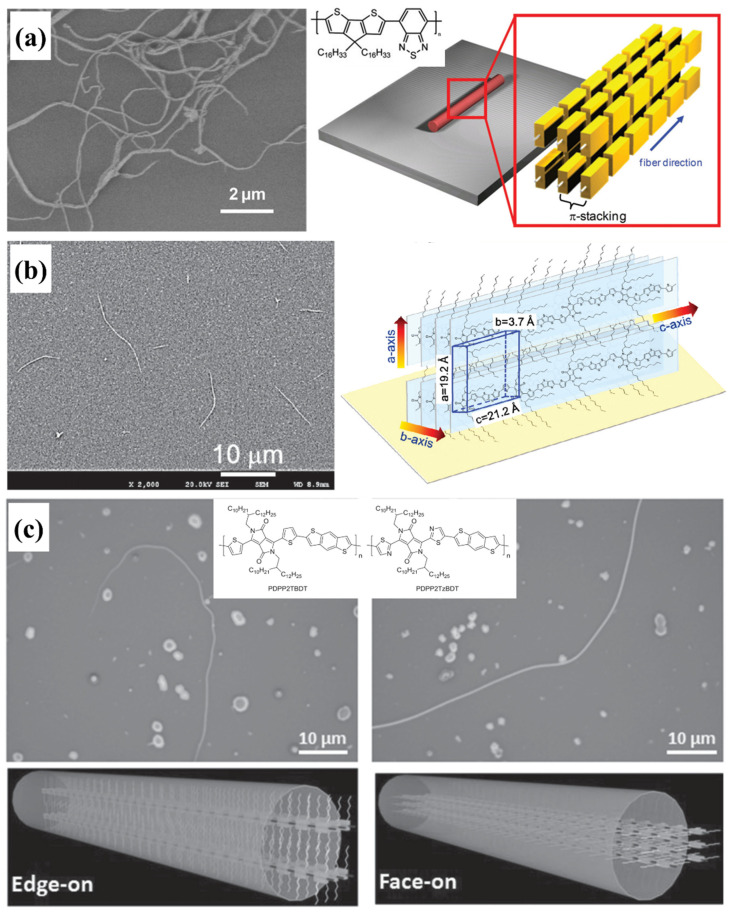
(**a**) Scanning electron microscopy (SEM) image of CDT-BTZ nanowires and schematic illustration of the backbone organization in the nanowires, which were adapted from reference [145] with permission from Copyright 2012 Wiley. (**b**) SEM image of PDTTDPP nanowires and schematic illustration of the proposed orientation of polymer chains in the nanowires, which were adapted from reference [82] with permission from Copyright 2013 Wiley. (**c**) Optical microscope (OM) images of the nanowires of the two PDPPs and schematic illustration of the molecular packing in the nanowires, which were adapted from reference [81] with permission from Copyright 2015 Wiley.

D-A conjugated polymer nanowires were first reported by Müllen et al., as shown in Figure 12a. They used the solvent vapor-enhanced drop-casting (SVED) method to promote the self-assembly of the cyclopentadithiophene-benzothiadiazole copolymer (CDT-BTZ). The nanowires adopted an edge-on orientation, and the length direction was along the backbone. The width, height and length of CDT-BTZ nanowires were 300–600 nm, 80–150 nm and 5–20 μm, respectively [145]. Later, Choi et al., prepared Poly[[2,5-bis(2-octyldodecyl)-2,3,5,6-tetrahydro-3,6-dioxopyrrolo[3,4-c]pyrrole-1,4-diyl]-alt-[[2,20-(2,5-thiophene)bis-dithieno(3,2-b;20,30-d)thiophene]-5,50-diyl]] (PDTTDPP) through self-assembly in a dilute solution. The nanowires also adopted an edge-on orientation with the length direction along the backbone as shown in Figure 12b [82]. They proposed that a more rigid and planar backbone could promote intermolecular interactions to propel the self-assembly of polymer chains, and, thus, it is beneficial for the formation of nanowires [80,82]. After that, Han et al., prepared PDBT nanowires using a method named slow evaporation of the main solvent (SEOMS). They proposed that decreasing the aggregate speeds of the molecules was very important to prepare D-A conjugated polymer nanowires because the growth of nanowires required that the polymer chains diffused to the growth front of the crystals [112]. According to the D-CT theory, the formation of nanowires required high *v*_D_/*v*_A_ and *v*_T_/*v*_A_ values [117]. A more rigid and planar backbone resulted in a higher *v*_T_ value. Decreasing the aggregate speeds meant a lower *v*_A_ value. Therefore, the views of both Choi et al. and Han et al., are critical for the preparation of nanowires. Besides the edge-on orientation, D-A conjugated polymer nanowires may also adopt a face-on orientation as shown in Figure 12c. Wang et al., prepared two PDPP (named PDPP2TBDT and PDPP2TzBDT) nanowires through an in situ drop-coating method. They found that PDPP2TBDT adopted an edge-on orientation, while PDPP2TzBDT adopted a face-on orientation [81]. In addition, the width and height of nanowires are generally less than 1 μm. If the width and height of nanowires are larger than 1 μm, the crystals could be called microwires. For example, Pei et al., prepared D-A conjugated polymer microwires by well controlling the nucleation and growth processes [135,146].

### 5.4. Rhizoid Crystals

**Figure 13:**
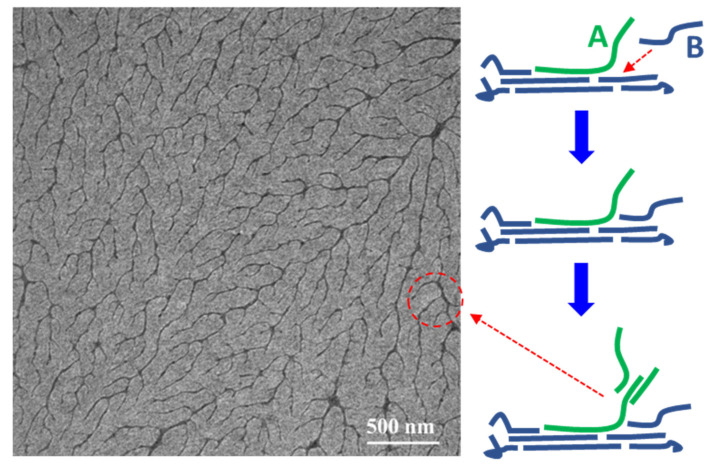
Schematic diagram of the formation process of rhizoid crystals, which was adapted from reference [117] with permission from Copyright 2022 Elsevier Ltd.

Branched fibrils first caught our group’s attention in 2021 [113]. We found that the morphology of pure PDQT films showed a branched fibril structure as shown in Figure 6a. The branched fibrils were regarded as dendritic crystals or dendritic fibrils at that time [113]. However, the formation of dendritic crystals happens through a diffusion-limited aggregation mechanism, which could not be used to explain the formation of branched fibrils [147,148,149]. We proposed that secondary nucleation might occur in the cilia of the fibril surface, and, thus, the branched structure was formed [113]. In fact, the nanowires of high-MW DPPBTSPE reported by Choi et al., also showed a branched structure as shown in Figure 6b [80]. However, this branched structure has received little attention. Our group named branched fibrils and nanowires as rhizoid crystals, and the D-CT theory could be used to explain the formation of rhizoid crystals as shown in Figure 13. Chain A diffuses to the growth front of the crystal, but it has not completed the conformational transition process. At the same time, chain B diffuses to the growth front and prevents the conformational transition of chain A. Then, the uncrystallized part of chain A may act as a new nucleus in the following growth process. As a result, a branched structure is formed. To accomplish the above processes, the crystallization conditions should satisfy *v*_D_ ≫ *v*_A_ ***≈*** *v*_T_ [117].

## 6. Conclusions and Outlook

We attempted to achieve a clearer understanding of the crystallization of D-A conjugated polymers in this review. First, the features of D-A conjugated polymers are the origin of their distinctive crystallization characteristics: (1) the geometrical regularity of the molecular structure determines the crystallizability of D-A conjugated polymers; (2) the strong backbone rigidity results in a typical extended chain conformation in the crystals; (3) the anisotropic interchain interactions result in a typical orthorhombic crystal unit cell, and the strong intermolecular π-π stacking interactions may manipulate the crystallization process; and (4) the alkyl side chains may self-crystallize or affect the crystallinity of the backbones. Then, the chain conformation in the solution as well as the nucleation and growth processes are key factors affecting the crystallization process. In solution, short-range ordered aggregates may act as nuclei, while unimer conformations are critical for the growth process, as they can reach the growth front of small crystals to form larger crystals. Adjusting the relative content of short-range ordered aggregates is a good way to control the nucleation process. In the growth process, the growth of crystals is connected to the *v*_D_, *v*_A_ and *v*_T_ values. Finally, the formation of multilevel structures in D-A conjugated polymer films largely depends on the crystallization process. The nucleation process and the orientation of the nuclei on the substrate are critical for the crystal structure. The resulting crystal morphology is a combined effect of the nucleation and growth processes.

Although many crystallization characteristics of D-A conjugated polymers can be well understood, the crystallization mechanism of D-A conjugated polymers is still not very clear. On the one hand, it is a challenge to describe the nucleation process. Although experimental results show that many factors, such as temperature, concentration and solvent additives, affect the nucleation process, a theoretical description of the nucleation process is absent. On the other hand, a quantitative description of the growth process is also a challenge. Therefore, further work is needed to better understand the crystallization of D-A conjugated polymers.

## Data Availability

The data presented in this study are available on request from the corresponding author.
